# Carbon-Based Modification Materials for Lithium-ion Battery Cathodes: Advances and Perspectives

**DOI:** 10.3389/fchem.2022.914930

**Published:** 2022-06-08

**Authors:** Luozeng Zhou, Hu Yang, Tingting Han, Yuanzhe Song, Guiting Yang, Linsen Li

**Affiliations:** ^1^ School of Chemistry and Chemical Engineering, Shanghai Jiao Tong University, Shanghai, China; ^2^ State Key Laboratory of Space Power-sources Technology, Shanghai Institute of Space Power-Sources, Shanghai, China; ^3^ School of Chemistry and Chemical Engineering, Nantong University, Nantong, China

**Keywords:** carbon-based materials, interfacial engineering, cathode materials, synthetic strategies, lithium-ion batteries

## Abstract

Lithium-ion batteries (LIBs) have attracted great attention as an advanced power source and energy-storage device for years due to their high energy densities. With rapid growing demands for large reversible capacity, high safety, and long-period stability of LIBs, more explorations have been focused on the development of high-performance cathode materials in recent decades. Carbon-based materials are one of the most promising cathode modification materials for LIBs due to their high electrical conductivity, large surface area, and structural mechanical stability. This feature review systematically outlines the significant advances of carbon-based materials for LIBs. The commonly used synthetic methods and recent research advances of cathode materials with carbon coatings are first represented. Then, the recent achievements and challenges of carbon-based materials in LiCoO_2_, LiNi_x_Co_y_Al_1-x-y_O_2_, and LiFePO_4_ cathode materials are summarized. In addition, the influence of different carbon-based nanostructures, including CNT-based networks and graphene-based architectures, on the performance of cathode materials is also discussed. Finally, we summarize the challenges and perspectives of carbon-based materials on the cathode material design for LIBs.

## Introduction

Over the past decades, lithium-ion batteries (LIBs) have a wide range of applications due to their great advantages of low cost, long cycling lives, and high-energy densities, including portable electronic devices, electric vehicles, and grid energy storage (Okubo et al., 2010; Cui et al., 2016). In LIBs, lithium-based metal oxides act as a cathode and graphitic carbon acts as an anode, and the electrolyte is a liquid organic solvent containing lithium salt (Nara et al., 2019). As the important components of LIBs, cathode materials play a key role in electrochemical performance (Nitta et al., 2015). Up to now, many cutting-point cathodes have been successfully commercialized, such as LiCoO_2_, LiNi_x_Co_y_Al_1−x−y_O_2_, and LiFePO_4_ cathodes (Li et al., 2020; Lu et al., 2020; Li et al., 2021a; Arabolla Rodríguez et al., 2021), promising LIBs a prominent position in the energy storage market. However, the cathode materials still face great challenges in practical applications, including slow charging, safety hazards, and high cost.

To solve these aforementioned problems, considerable efforts have been devoted to developing the following characteristics: 1) high ionic conductivity and Li diffusion coefficient; 2) high redox potential; 3) stable structure; 4) superior corrosion resistance; and 5) low cost (Du et al., 2019; Chakraborty et al., 2020; Logan and Dahn, 2020; Wu et al., 2020). Carbon-based materials like carbon nanofibers (CNFs) (Chen et al., 2022), carbon nanotubes (CNTs) (Li et al., 2021b; Chen et al., 2022), graphene (Chang et al., 2015), and their composites have been considered as crucial modifying agents owing to their outstanding properties, contributing to the improvement as follows: low cost, special structure, tunability, good dispersion, and stability. Moreover, the types of carbon-based materials show great differences in the dimensionality, morphology, and distribution of chemical bonding compared to traditional materials, which involve in the mixtures of local electronic structures between sp^2^ and sp^3^ (Palshin et al., 1995). As a result, fabricating cathode materials with carbon matrix can improve their conductivity and mitigate the volume variation.

In this review, we first summarize various strategies by using carbon-based materials to improve the properties of cathodes and their applications in LIBs. Furthermore, carbon-based materials in LiCoO_2_, LiNi_x_Co_y_Al_1−x−y_O_2,_ and LiFePO_4_ cathodes are discussed and summarized. Finally, the advantages and perspectives of the cathodes are also discussed.

## Fundamentals and Challenges of Cathode Materials

**FIGURE 1:**
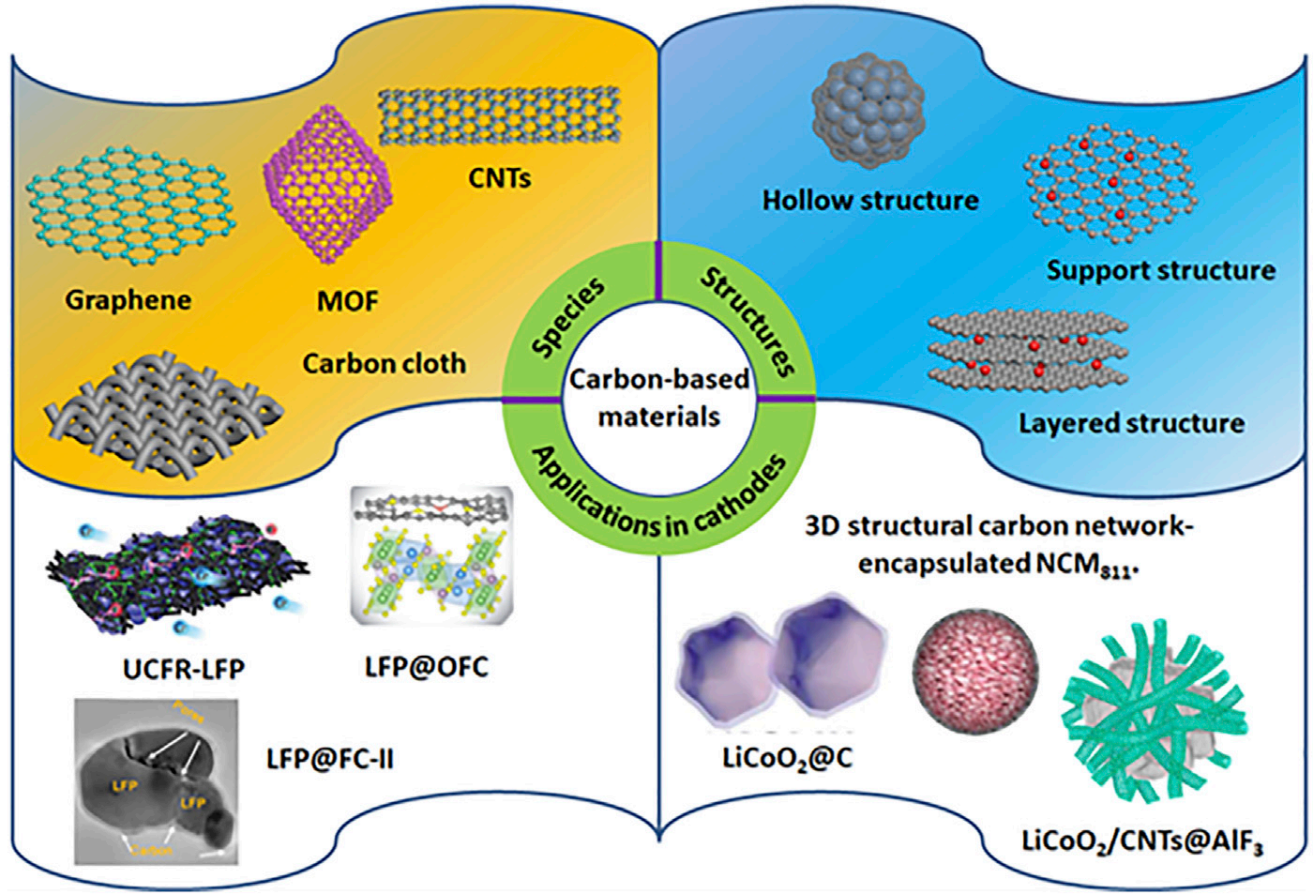
Schematic diagram of carbon-based materials in addressing the challenges of LIBs in different cathodes. Reprinted with permission from Li H. et al. (2019) with permission from WILEY-VCH. Reprinted with permission from Lin J. et al. (2022), Wang X. et al. (2019), and Hwang. et al. (2020) with permission from Elsevier. Reprinted with permission from Lin J. et al. (2020) with permission from The Royal Society of Chemistry. Reprinted with permission from Cheng Q. et al. (2019) with permission from the American Chemical Society.

The charge/discharge process in LIBs belongs to a chemical process of Li intercalation and deintercalation. The charge progress makes the cathode lithium deficient and the anode lithium rich, while the discharge process runs the other way. However, the structure of lithium-based metal oxides determines the charge/discharge rate and specific capacity compared to graphite anode. The cathode materials can be divided into the following types based on their crystal structures: layered structure, spinel structure, and olivine structure. Despite the advances, they still suffer from poor electrical conductivity, slow Li transport, unfavorable interactions with the electrolyte, low thermal stability, and high-volume expansion (Sun et al., 2016; Liu et al., 2018; Yuan et al., 2021). To solve the issues, various methods have been developed, including tuning morphology and structure, doping metallic cation, and integrating them with conductive carbon materials. Among them, it is an effective way to circumvent the problems by using conductive carbon materials to modify the cathode materials, such as constructing carbonaceous composites, doping carbon-based materials on cathodes, and coating carbon-based materials. In contrast, the advantages of the coating methods involve in providing good electrical conductivity, hindering the cathode dissolution and electrolyte degradation, and shortening the length of Li^+^ diffusion. As a result, to shed some light on the progress in cathodes, we summarize the recent reports of emerging carbon-based materials for various cathode materials by discussing their performance and underlying mechanisms (Figure 1).

## Carbon-Based Materials for the Layered LiCoO_2_ Cathode

Layered LiMO_2_ (M = Co, Mn, Ni) oxides have been considered one of the most common cathode materials for LIBs. LiCoO_2_ is first employed as the intercalation cathode for LIBs by Goodenough, Mizushima et al. (1980), and Kubota (2020). The LiCoO_2_ cathode has the advantages of high theoretical specific capacity (274 mAh g^−1^), high theoretical volumetric capacity (1,363 mAh cm^−3^), high discharge voltage, and good cycling performance. However, the reversible intercalation/deintercalation amount of Li^+^ is only 0.5 units since the layered-structure collapse by LiCoO_2_ lattice distorting from hexagonal to monoclinic symmetry when discharge takes place deeply. In addition, LiCoO_2_ would emit oxygen during the charging process at high temperatures, which leads to volume change, resulting in a lower capacity.

It has been reported numerous efforts to enhance the LiCoO_2_ by using advanced materials, especially carbon-based materials. Wang reported the sandwich-structured LiCoO_2_ cathodes by using cross-stacked super-aligned carbon nanotubes (SACNTs) (Yan et al., 2017). The cathodes consist of a repeating and alternating stack of LiCoO_2_ layers and SACNT films, in which each layer of active materials could adhere to the SACNT conductive layers, realizing sufficient electron transfer throughout the electrodes. The sandwiched LiCoO_2_ cathode could deliver a specific discharge capacity of 109.6 mAh g^−1^ at 10C, demonstrating much higher energy densities. In addition, ZIF-67 precursors were annealed with Li_2_CO_3_ under air and followed by homogeneous AlF_3_ coating and carbon nanotubes wrapping, which can provide rapid internal electron channels and the external surface AlF_3_-coating (Cheng et al., 2019). Therefore, AlF_3_-coated LiCoO_2_/CNTs can enhance lithium storage performance at both room temperature and an elevated temperature (50°C) and illustrate an excellent rate and cycling performance with 120 mAh g^−1^ at 1C after 200 cycles. It can be seen that CNT materials not only lower the energy barrier but also improve the rate performance (Table 1).

**TABLE 1:** Electrochemical performance of carbon-based materials that modified cathodes.

**Cathode material**	**Initial capacity**	**Final capacity**	**Cycle**	**Ref**
LiCoO_2_-CMF	191.1 mAh g^−1^	180.1 mAh g^−1^	300 (0.5 C)	Lu et al.
LiCoO_2_/CNTs@AlF_3_	134.9 mAh g^−1^	120 mAh g^−1^	200 (1 C)	Cheng et al.
LiF-C@ LiCoO_2_	181 mAh g^−1^	161 mAh g^−1^	180 (0.5 C)	Lim et al.
N-doped LiCoO_2_@C-700	—	171.1 mAh g^−1^	200 (1 C)	Lin et al.
NCACS	178 mAh g^−1^	150 mAh g^−1^	250 (0.5 C)	Vadivel et al.
NCA-LB1	171.7 mAh g^−1^	160.7 mAh g^−1^	100 (2 C)	Du et al.
LPAN@NCA	227.9 mAh g^−1^	213.3 mAh g^−1^	200 (0.1 C)	Gui et al., 2019
LNCA-VACNTs	—	153.6 mAh g^−1^	120 (1 C)	Tian et al., 2020
C-LFP-S	139 mAh g^−1^	116 mAh g^−1^	1,000 (10 C)	Hwang et al.
UCFR-LFP	149.5 mAh g^−1^	108 mAh g^−1^	315 (2 C)	Li et al.
LFP@OFC	—	160.9 mAh g^−1^	500 (1 C)	Lin et al.
LFP@FC-II	122.6 mAh g^−1^	106.2 mAh g^−1^	1,000 (10 C)	Wang et al.
LFP/C@CMK-8	120 mAh g^−1^	116 mAh g^−1^	1,000 (10 C)	Saikia et al.

Su designed an *in-situ* modification strategy to synthesize a metal–organic framework (MOF)-derived LiCoO_2_ heterostructure (Lin et al., 2020). The LiCoO_2_@C cathode shows long cycling life (171.1 mAh g^−1^ at 1C after 200 cycles) and superior rate capability (150.3 mAh g^−1^ even at 10C). Both experimental and DFT results prove that an *in-situ* MOF-derived carbon-coating strategy can not only reduce direct contact resistance between the LiCoO_2_ bulk and electrolyte but also boost the electrochemical conductivity and strain structural distortion, resulting in enhanced cyclability and rate performance. In addition, the low energy barriers of Li-vacancy migration pathways on the LiCoO_2_@C heterostructure are helpful to build a systematic diffusion network. Lee found that the LiF-C layer formed on LiCoO_2_ by defluorination reaction with carbon monofluoride at 400°C can suppress side reactions at the electrolyte/electrode interface (Lim et al., 2021). As a result, the LiCoO_2_ cathode with the LiF-C layers could deliver an initial discharge capacity of 186 mAh g^−1^ at 0.1C and achieve a long-term cycle at 4.5 V. Importantly, the modified LiCoO_2_ cathode indicates excellent cycling and rate performance, which delivers 161 mAh g^−1^ after 180 cycles and 115 mAh g^−1^ at 10C.

In general, the structural stability and cycle performance of LiCoO_2_ can be greatly improved by carbon-based materials at high voltage operation (>4.2 V). Constructing carbon-based coating is a promising synthetic approach to enhance the structural property and electrochemical performance of LiCoO_2_ cathode. The main contribution of carbon-based materials is to efficiently improve the electrical conductivity within the cathode host and reduce the direct contact resistance between the LiCoO_2_ bulk and electrolyte, leading to the extensive application of LiCoO_2_ cathodes in LIBs with good rate performance and cycling life.

## Carbon-Based Materials for the Layered LiNi_x_Co_y_Al_1-x-y_O_2_ Cathode

The further massive usage of LiCoO_2_ cathodes for LIBs has been severely hindered by the sharp rising cost and limitation of cobalt resources. Considerable works have been committed to developing less Co or Co-free cathode materials, such as LiNiO_2_, LiNi_0.8_Co_0.2_O_2_, and LiNi_x_Co_y_Al_1-x-y_O_2_. Until now, the most prospective alternative for LiCoO_2_ cathode is LiNi_x_Co_y_Al_1-x-y_O_2_ (NCA) due to its affordable cost, high-energy density, and structural stability (Liu et al., 2015; Zheng et al., 2017). Tesla company first employs NCA cathodes to drive cars, and it has achieved remarkable success in the electric vehicle industry. However, Ni^2+^ ions could easily occupy the vacancy of Li^+^ ions during the charging process, which may increase the degree of cation mixing and generate irreversible phases, resulting in capacity loss. In addition, the thermal stability of NCA should be promoted for the reduction of unstable Ni^3+^ at high temperatures, which would destroy the layered structure.

To overcome the shortages of NCA, the modification of carbon-based materials can effectively enhance ionic conductivity and cycle performance by changing the transmission mechanism and increasing the electrical conductivity. Many approaches have been developed to coat LiNi_x_Co_y_Al_1-x-y_O_2_ by using carbon-based materials, including atomic layer deposition (ALD) (Gupta et al., 2022), wet chemical method (Han et al., 2019), and chemical vapor deposition (CVD). The hydrolysis–condensation method was designed to synthesize the NCA-ethoxy-functional groups (EPS) cathodes, which can effectively suppress the side reactions and mitigate the instability of pristine NCA (Visbal et al., 2016). The cycling stability of the NCA-EPS cathode was significantly improved, in which EPS as a protective shell can reduce the trace water on the secondary particle surface and inhibit the side reaction. Visbal et al. (2016) used a CVD approach to investigate the impact of diamond-like carbon (DLC) layers on LiNi_0.8_Co_0.15_Al_0.05_O_2_ materials. The chemical inertness of the DLC coating can contribute to preventing unwanted reactions between the cathode active material and sulfide solid electrolyte. That is because the DLC layers consist of sp^3^ and sp^2^ carbon structures, which have superior properties, including high hardness, chemical inertness, and high thermal conductivity.

A solvent-free and economically feasible process was designed by using electrically conductive nanocarbon, which can produce LiNi_0.8_Co_0.15_Al_0.05_O_2_-carbon composites (Vadivel et al., 2019). LiNi_0.8_Co_0.15_Al_0.05_O_2_-carbon compounds could keep their initial structure under the high-speed operation (∼5,000 rpm) and display high reversible capacity and cycle performance (91% at 0.1C and 84% retention at 0.5C after 250 cycles). Interestingly, exfoliated graphene nanosheets that were functionalized with an amphiphilic surfactant were employed as the coatings (Park et al., 2021). The graphene nanosheets can attach onto Ni-rich oxides in a face-to-face manner owing to the adhesive amphiphilic surfactant. By eliminating the conventional conductive additive and minimizing the binder content, the graphene-coated electrode demonstrates a highly dense 99 wt% NCA (electrode density ∼4.3 g cm^−3^) with a high areal capacity of ∼5.4 mAh cm^−2^ (∼38% increase) and high volumetric capacity of ∼863 mAh cm^−3^ (∼34% increase) at a current rate of 0.2 C (∼1.1 mA cm^−2^), relative to the bare electrode with a commercial level of 96 wt% NCA (electrode density, ∼3.3 g cm^−3^).

As the prospective cathode of LIBs, it should pay more attention to the performance degradation and safety hazard of NCA materials, which are raised from aggressive chemical, structural, and mechanical deterioration and thermodynamic instability. The carbon-based coating can effectively improve the ion/electron conductivity and cycle performance of NCA materials by increasing the electronic transport pathways and changing the transmission mechanism. Therefore, the carbon-based coating can protect the structure of NCA particles from collapsing by puzzled side reactions and remarkably improve the stability of the battery during cycling.

## Carbon-Based Materials for the Olivine-Type LiFePO_4_ Cathode

LiFePO_4_ belongs to the lithium ortho-phosphates with an orthorhombic lattice structure, which takes many advantages, including low cost, thermal stability, and environmental compatibility. However, the slow intrinsic electronic conductivity, poor Li^+^ diffusion coefficient, and relatively low theoretical capacity obstruct their applications in high-energy-density fields. Carbon-based materials can increase electron migration and Li^+^ transfer and prevent particle aggregation, which have become the crucial materials to improve electrode properties.

To realize excellent rate performance, LiFePO_4_/reduced graphene oxide (RGO) hybrid was synthesized via a homogeneous coprecipitation method, which demonstrates a specific capacity of 172 mAh g^−1^ at 0.06C and 139 mAh g^−1^ at 11.8C (Zhu et al., 2014). LiFePO_4_ particles are uniformly and closely anchored upon conductive RGO sheets, which improve electron/ion transmission and prevent LiFePO_4_ particle aggregation. Ultrathin conformal carbon was coated on LiFePO_4_ nano/microspheres by using liquid carbon dioxide, exhibiting good reversible capacity (168 mAh g^−1^ at 0.1C), high energy density, and excellent long-term cyclability (84% cycle retention at 10C after 1,000 cycles) (Hwang et al., 2017). The average thickness of the carbon layer distributed over the primary LiFePO_4_ particles is 1.9 nm, providing facile penetration of liquid electrolytes and a rapid ion/electron transmission channel. Moreover, the high tap density (1.4 g cm^−3^) of the ultrathin carbon layer contributes to a high volumetric energy density (458 Wh L^−1^ at a 30C rate).

Fluorine-doped carbon (FC) materials are introduced to coat the LiFePO_4_ cathode by using polyvinylidene fluoride. A three-dimensional conductive network structure was obtained, in which FC is observed to attach to the LiFePO_4_ cathode (Wang et al., 2020). The LiFePO_4_ cathode with FC coating can deliver an attractive cycling stability over 1,000 cycles and possess a high-rate capability with maintaining 100.2 mAh g^−1^ at 20C, due to the shortening of Li^+^ diffusion distance and the rapid transfer of electrons. Moreover, C-F covalent bonds improve the strong chemical affinity between the electrolyte and LiFePO_4_ electrode because of their excellent permeation in the electrolyte. Lin et al. (2022) reported versatile LiFePO_4_ microparticles encapsulated in an O, F-codoped carbon matrix (LFP@OFC) via solid-state sintering. The O, F-codoped porous carbon matrix is energetically preferable for superior adsorption capability coupled with decreased diffusion barriers of Li^+^, contributing to abundant active sites, boosted electronic conductivity, and expedited diffusion aisles. Therefore, LFP@OFC could deliver a specific capacity (169.9 mAh g^−1^ at 0.1C) and have rate capacity (85.6 mAh g^−1^ even at 16.2C) and long-term cyclability (160.9 mAh g^−1^ over 500 cycles at 1C).

LiFePO_4_ has been widely acknowledged for its poor electronic conductivity (∼10^−9^ S cm^−1^) and low Li^+^ diffusion coefficient (∼10^−14^ cm^2^ s^−1^). To improve its capacity and rate performance, many approaches have been proposed with organic or inorganic carbon sources, such as citric acid, lactose and glucose, super P, carbon nanotubes (CNTs), and graphene. The basic purpose of the carbon-based coating is to cover LiFePO_4_ particles with a uniform conductive layer to improve the surface conductivity and utilize the active material at high rates. In addition, carbon coating can also prevent side reactions at the electrode–electrolyte interface and restrict the growth of the crystal.

## Conclusion and Perspectives

In summary, carbon-based materials have been widely used due to their excellent stability and high electronic conductivity in LIBs. The electrochemical performance of typical cathodes has been greatly improved due to the protective effect of carbon coating. Carbon-based coatings not only construct the conductive ion/electron transfer path between active cathodes and electrolytes but also play an important role in restricting the cathode dissolution and electrolyte degradation. Therefore, the primary purpose of carbon-based materials on the surface of the LiCoO_2_ cathode is to stabilize the crystal structure at high-voltage (over 4.2 V) cycling, inhibiting the detrimental phase transition from the hexagonal structure to monoclinic structure. The carbon-based materials can prevent the crystal structure of LiNi_x_Co_y_Al_1-x-y_O_2_ from collapsing due to the side reaction since the poor thermal stability of Ni^3+^ in a lattice structure is extremely unstable under high temperature and easily reactive with the HF. The most important improvement of carbon-based materials for LiFePO_4_ is to increase its intrinsic slow Li-ion diffusion and electronic conductivity, especially for high-rate performance.

Despite the great success of LIBs, the dramatic increase of energy-density need still promotes the fast development of cathode materials with superior specific capacity and affordable cost. As originally commercialized by SONY in the 1990s, LiCoO_2_ will continue its great success in the consumer electronic device industry if breaking through its practical energy density by further elevating charge cut-off voltage higher than 4.5 V. Meanwhile, LiNi_x_Co_y_Al_1-x-y_O_2_ may play an important role in the practical fields of high-energy density. With consideration of its outstanding advantages in safety performance and thermal stability, LiFePO_4_ could be predicted to dominate the application market where prioritizing security and cost among the major commercialized cathodes. Carbon-coating engineering has been extensively studied to separate cathodes from electrolyte solutions and improve conductivity. It is also essential to take into consideration the compatibility of the modified cathode with the electrolyte and anode. To satisfy the demands of large-scale production, the modification process should avoid the usage of energy-intensive methods. In addition, carbon-based materials should be obtained easily from a wide range of sources. Undeniably, there is still a huge development potential for persistent exploration of carbon-based materials as a breakthrough research direction for further pursuing the higher energy density, better property, and longer cycle life of batteries.
